# Comparative analysis of corticosteroid injection alone versus combined corticosteroid and radiofrequency ablation for plantar fasciitis: short- and medium-term outcomes

**DOI:** 10.55730/1300-0144.6350

**Published:** 2026-04-28

**Authors:** Nevcihan ŞAHUTOĞLU BAL, Emel BAŞAR, Hatice BABAOĞLAN, Mustafa Cem YILMAZ, Ülkü SABUNCU, Şükriye DADALI, Erkan Yavuz AKÇABOY

**Affiliations:** Department of Pain Medicine, Ankara Bilkent City Hospital, Ankara, Turkiye

**Keywords:** Corticosteroid injection, heel pain, pain measurement, plantar fasciitis, radiofrequency ablation, treatment outcome

## Abstract

**Background/aim:**

Plantar fasciitis (PF) is a common cause of heel pain and may lead to functional impairment and reduced quality of life. This study aimed to compare the short- and medium-term clinical outcomes of corticosteroid injection (CSI) alone versus combined radiofrequency ablation (RFA) and CSI in patients with PF refractory to conservative treatment.

**Materials and methods:**

This single-center retrospective comparative study included patients diagnosed with PF between 1 January 2021 and 1 January 2025. Patients who underwent either CSI alone or combined RFA and CSI after failure of conservative management were included. Pain intensity was assessed using a visual analog scale (VAS) at baseline, 1 week, 3 months, and 6 months. Global perceived effect (GPE) scores were evaluated at 1 week, 3 months, and 6 months.

**Results:**

Both treatment groups demonstrated significant improvements in VAS scores at 1 week, 3 months, and 6 months compared to baseline (p < 0.001 for all). Baseline and 1-week VAS scores were comparable between the groups (p > 0.05). However, median VAS scores at 3 and 6 months were significantly lower in the group receiving combined RFA and CSI compared to CSI alone (p < 0.001). GPE scores at 1 week did not differ significantly (p = 0.100), but at 3 and 6 months, they were significantly higher in the combined group (p < 0.001). In adjusted longitudinal analysis, the group receiving combined RFA and CSI had lower overall VAS scores over time, with significant group × time interactions at 3 and 6 months.

**Conclusion:**

Both CSI alone and combined RFA and CSI provide significant short- and medium-term pain relief. However, the addition of RFA to CSI is associated with superior midterm pain control and greater patient-reported clinical improvement.

## 1. Introduction

Plantar fasciitis (PF) is the most common cause of heel pain and is characterized by inflammation and degeneration of the plantar fascia due to repetitive microtrauma. It can affect individuals of all activity levels but is particularly prevalent among middle-aged and older adults. The etiology of PF is multifactorial and often idiopathic. Internal risk factors include obesity, flat feet, high arches, and tight Achilles tendons, all of which alter foot biomechanics and increase the stress on the plantar fascia. External contributors include prolonged standing, walking barefoot on hard surfaces, and wearing unsupportive footwear [1]. Heel pain affects approximately 10%–15% of the adult population and can significantly impair mobility and quality of life [2].

Histopathological changes in PF primarily involve collagen fiber degeneration and fibroblastic tissue proliferation in response to chronic mechanical overload. In some cases, these changes are accompanied by soft tissue ossification at the calcaneal origin, forming a calcaneal spur. However, heel spurs are not always symptomatic, with approximately 15% remaining painless [3,4]. Chronic heel pain that does not resolve may become functionally disabling and resistant to conservative interventions.

Although various treatments have been proposed, including stretching exercises, physical therapy, and surgical options, no consensus exists regarding the optimal approach. Recently, percutaneous techniques have gained popularity as minimally invasive treatment options. Among these, radiofrequency ablation (RFA) has emerged as a promising modality for managing chronic musculoskeletal pain conditions, including PF and lateral epicondylitis [5–8]. It utilizes a high-frequency, low-voltage current to generate localized thermal energy, resulting in tissue coagulation and nerve denervation. The procedure not only disrupts pain transmission by targeting sensory nerve endings but may also reduce plantar fascia thickness by breaking down collagen bonds [9].

Despite the increasing use of RFA in the treatment of PF, evidence of its effectiveness in comparison to that of conventional approaches, such as corticosteroid injection (CSI), remains limited. In this context, we aimed to assess whether combining RFA with CSI provides superior short- and medium-term outcomes compared to CSI alone in patients with chronic PF unresponsive to conservative therapy.

## 2. Materials and methods

Thissingle-center retrospective comparative study included consecutive patients diagnosed with PF at a tertiary care hospital between 1 January 2021 and 1 January 2025. Patients who underwent either CSI alone or combined RFA and CSI after failure of conservative management were evaluated. The study protocol was approved by the relevant institutional ethics committee (Date: 02.01.2025, No: 1-25-873) and conducted in accordance with the Declaration of Helsinki. All participants provided written informed consent.

### 2.1. Patient selection

Data were collected from patients diagnosed with PF who had not improved after at least 6 months of noninvasive conservative treatments, including stretching exercises, nonsteroidal antiinflammatory drugs, weight management, lifestyle modifications, heel cups, arch supports, night splints, and platelet-rich plasma injections, and who subsequently received either combined RFA and CSI or CSI alone.

Eligible patients were categorized into two groups based on the treatment received, with one group receiving CSI alone and one group receiving combined RFA and CSI. Age, sex, pain scores, and global perceived effect (GPE) scores were compared between the groups. Visual analog scale (VAS) scores were recorded at baseline (preintervention) and at 1 week, 3 months, and 6 months after the intervention. GPE scores were assessed at 1 week, 3 months, and 6 months.

Participants were excluded from the study based on the following criteria: history of surgical intervention in the affected extremity; body mass index (BMI) greater than 30 kg/m^2^; previous fungal or other infections in the affected extremity; vascular pathology in the affected extremity; rheumatic diseases; mental disorders; neurological foot disorders, clubfoot, pes cavus, or pes planovalgus; or inaccessible medical records.

### 2.2. Outcome measures

The VAS is a visual measurement tool ranging from 0 to 10 cm, where 0 represents no pain and 10 represents the most severe pain imaginable [10]. The GPE score was used to reflect the patient’s subjective perception of change in the primary complaint [11,12]. Global perceived effect was assessed using a 7-point Likert-type scale with the following percentage-based interpretations: 7 = very much improved (≥75% improvement); 6 = much improved (50%–74% improvement); 5 = slightly improved (25%–49% improvement), 4 = no change (0%–24% change); 3 = slightly worsened (25%–49% worsening); 2 = much worsened (50%–74% worsening); 1 = very much worsened (≥75% worsening).

### 2.3. Procedure

Before the intervention, routine laboratory tests were conducted and peripheral vascular access was established. All procedures were performed under sterile conditions in an operating room equipped with standard anesthesia monitoring. The procedures were performed under fluoroscopic guidance, which was the standard clinical protocol in our department during the study period, providing reliable anatomical orientation through bony landmarks.

#### 2.3.1. Corticosteroid injection procedure

The patient was placed in the supine position, with the ankle of the affected extremity slightly rotated laterally from the neutral position. After disinfecting the skin with 10% povidone–iodine solution, local infiltration anesthesia was achieved using 2% prilocaine (1–2 mL) to the medial aspect of the calcaneus at the anatomical insertion of the plantar fascia under fluoroscopic guidance. Following adequate anesthesia, CSI was performed using 40 mg of methylprednisolone acetate into the targeted area.

#### 2.3.2. Radiofrequency ablation and corticosteroid injection procedure

**Figure 1:**
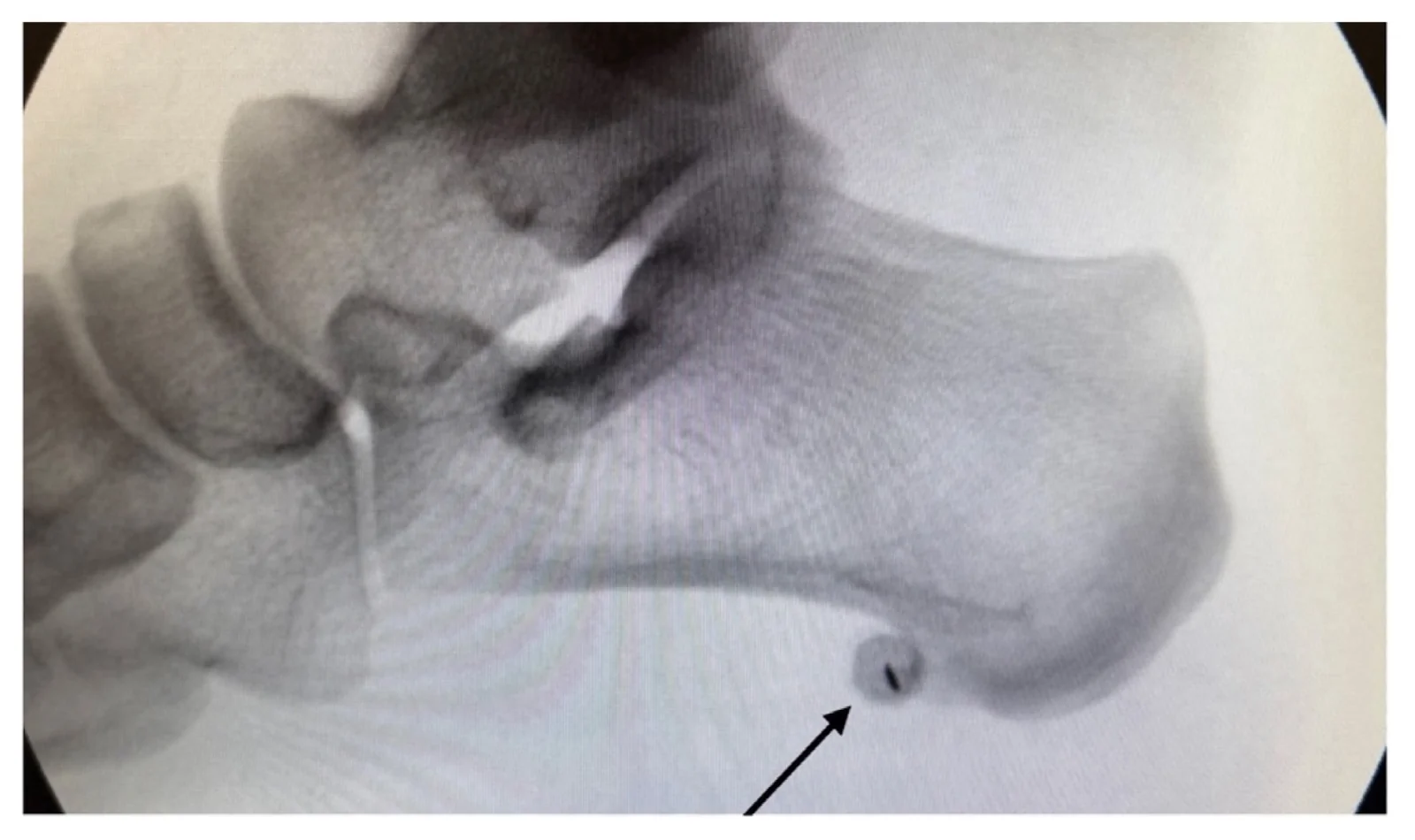
Fluoroscopic image showing the calcaneal spur and the positioned RFA electrode (arrow).

The patient was placed in the supine position and a grounding pad was applied to the contralateral extremity. Under fluoroscopic guidance, a 22-gauge radiofrequency cannula (5 cm in length, 5-mm active tip) and radiofrequency electrode were positioned adjacent to the medial calcaneal tuberosity as shown in Figure 1. Motor stimulation at 2 Hz and sensory stimulation at 50 Hz were used to confirm correct placement. Conventional thermal RFA was applied at 80 °C for 60 s. Immediately following ablation, CSI with 40 mg of methylprednisolone acetate was administered to the same area.

### 2.4. Statistical analysis

Recorded data were analyzed using IBM SPSS Statistics 27.0 for Windows (IBM Corp., Armonk, NY, USA). The normality of continuous variables was assessed using the Kolmogorov–Smirnov and Shapiro–Wilk tests. Normally distributed continuous variables were expressed as mean ± standard deviation, whereas nonnormally distributed continuous variables were presented as median (interquartile range [IQR], 25th–75th percentiles). Categorical variables were summarized as frequencies and percentages. Between-group comparisons were performed using the Pearson chi-square test for categorical variables, the independent-samples t-test for normally distributed continuous variables, and the Mann–Whitney U test for nonnormally distributed continuous variables. The false discovery rate was applied to adjust for multiple comparisons between groups where appropriate.

Within-group changes in repeated nonnormally distributed variables were initially evaluated using the Wilcoxon signed-rank test; however, longitudinal trends were primarily assessed using linear mixed-effects models. In addition, to account for repeated measurements over time and to assess the interaction between treatment group and time, a linear mixed-effects model was applied with treatment group, time, and group × time interaction as fixed effects, while the patient was a random effect and age and sex were included as covariates. Model assumptions were evaluated through residual diagnostics. The normality of residuals was assessed using Q–Q plots and a histogram. Estimated marginal means derived from the model were used to generate interaction plots. Effect size estimates (Cohen’s d) and 95% confidence intervals were reported where appropriate. A two-sided value of p < 0.05 was considered statistically significant. Post hoc power analysis was performed using open-source G*Power software (version 3.1.9.7). For the primary outcome (VAS score at 6 months), with an alpha level of 0.05 and a total sample size of 178, the statistical power was 0.94, confirming adequate power to detect significant differences between the treatment groups.

## 3. Results

A total of 178 patients were included in the study. Of these, 78 patients received CSI alone, while 100 patients underwent combined RFA and CSI. The demographic characteristics of these study groups are summarized in Table 1. The groups were comparable with respect to age and sex (p > 0.05).

**Figure 2:**
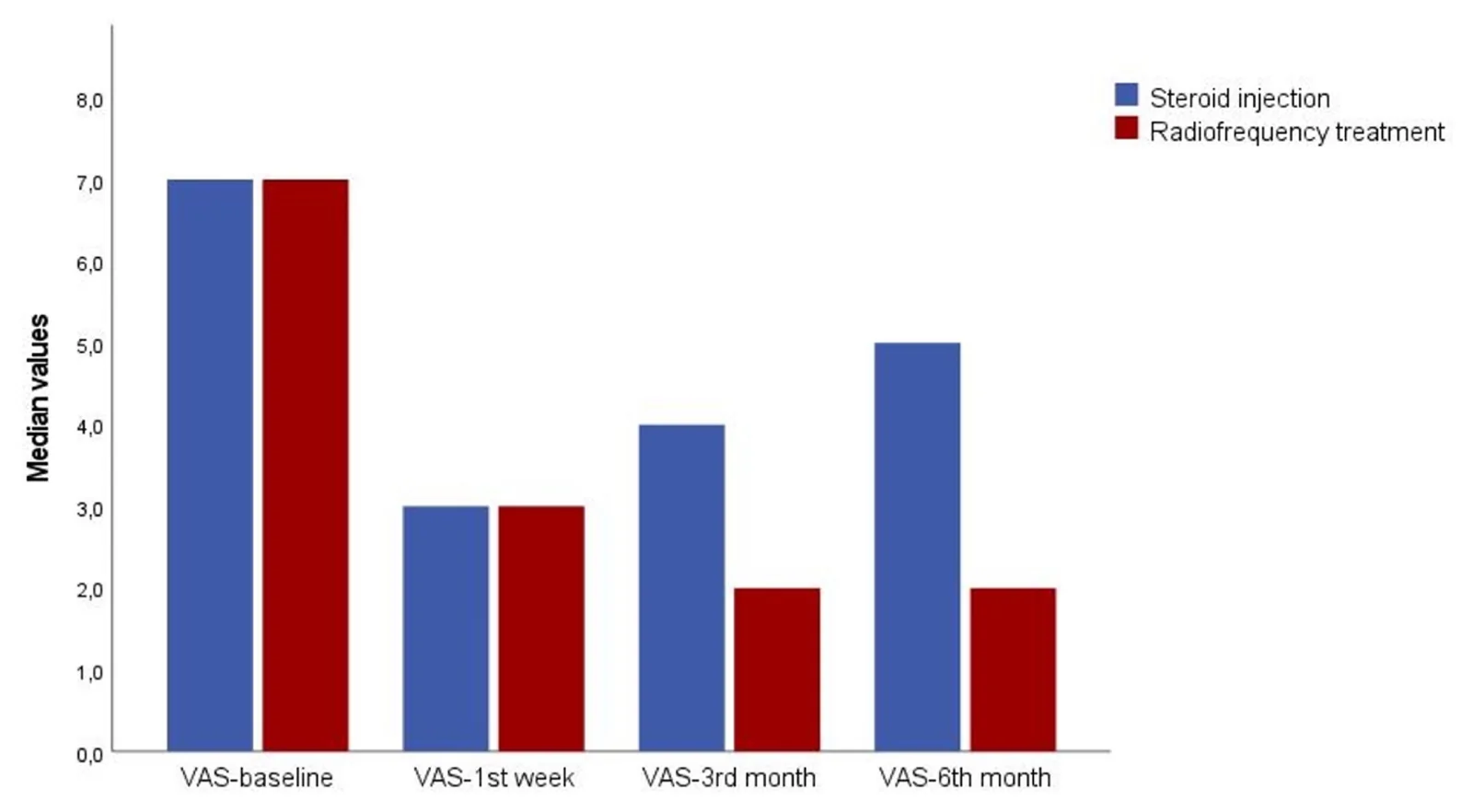
VAS scores of the groups at baseline and during follow-up.

**Figure 3:**
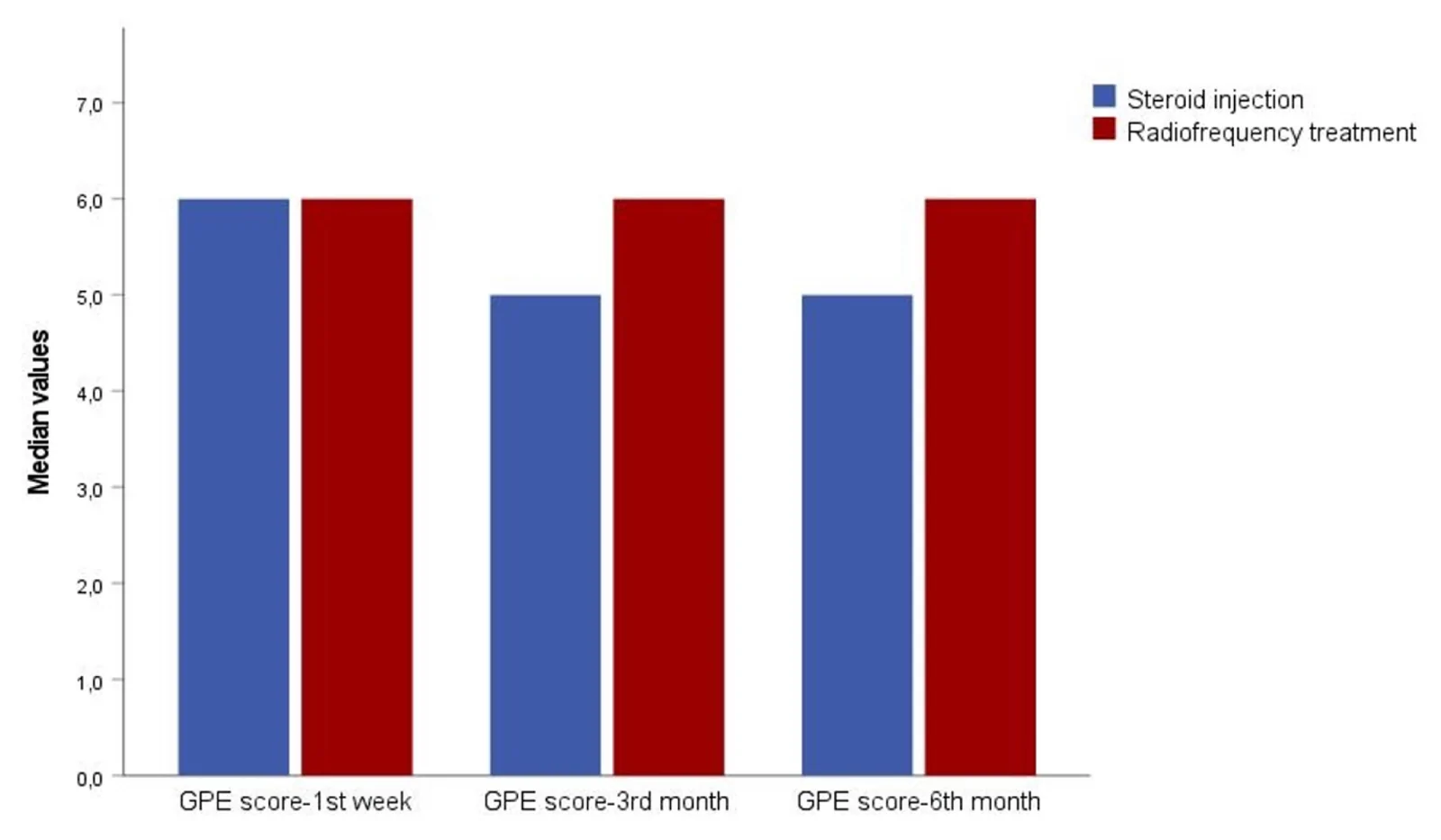
Global perceived effect scores of the groups.

Visual analog scale and global perceived effect scores during follow-up, as well as intra- and intergroup comparisons, are presented in Table 2. Both groups showed significant reductions in VAS scores at 1 week, 3 months, and 6 months compared to baseline values (p < 0.001 for all). No significant differences in VAS scores were observed between the groups at baseline or at 1 week (p > 0.05). However, median VAS scores at 3 and 6 months were significantly lower in the group receiving combined RFA and CSI compared to CSI alone (p < 0.001). GPE scores did not differ significantly between the groups at 1 week (p = 0.100). In contrast, GPE scores at both 3 and 6 months were significantly higher in the group receiving combined RFA and CSI (p < 0.001 for both time points). Trends in VAS and GPE scores over time are illustrated in Figures 2 and 3, respectively.

The proportion of patients achieving clinically meaningful pain relief, defined as ≥50% reduction in pain intensity, was similar between the groups at 1 week (p = 0.798), but was significantly higher in the group receiving combined RFA and CSI at 3 and 6 months (p < 0.001 for both comparisons) (Table 3).

**Figure 4:**
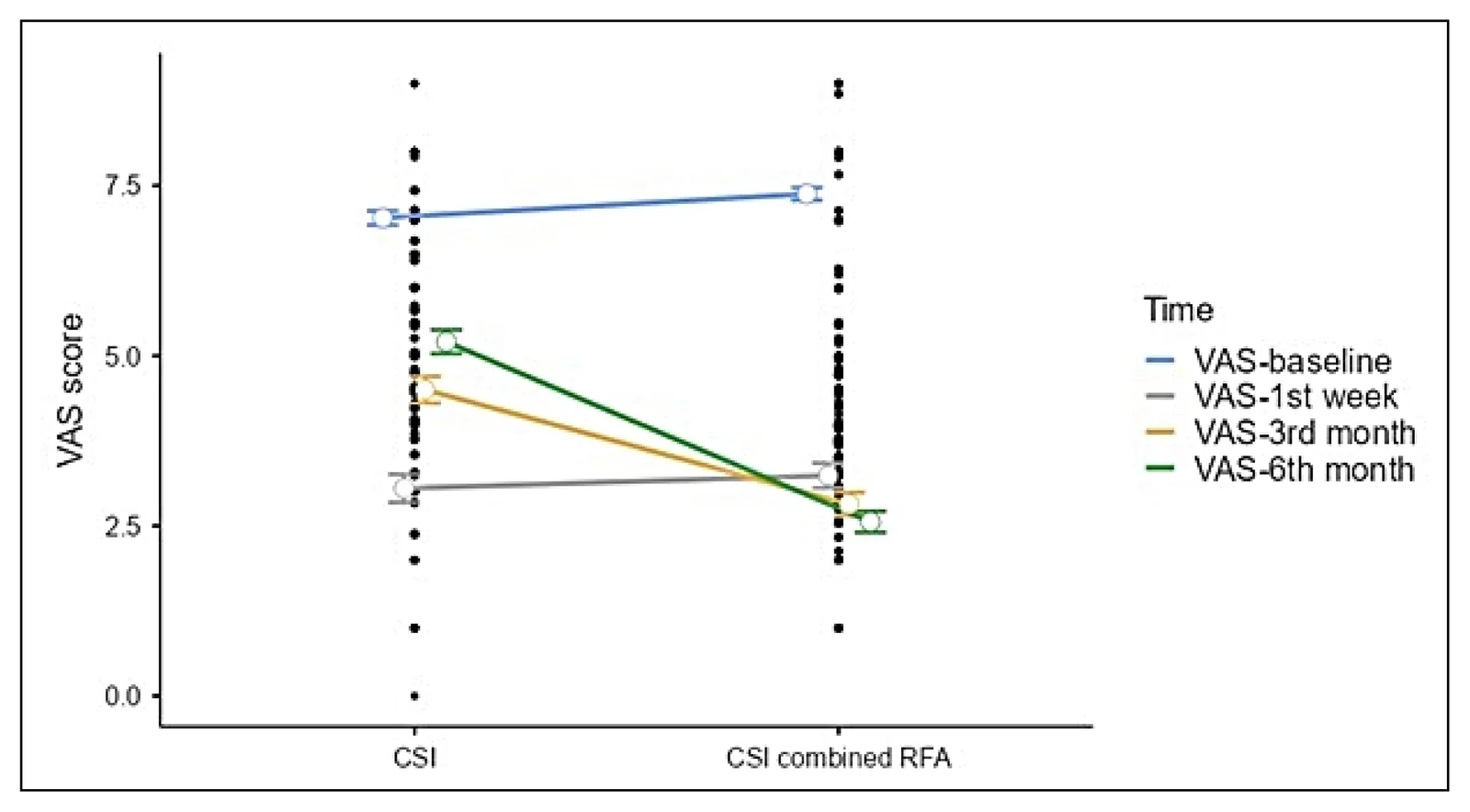
Interaction plot of estimated marginal means for VAS scores across time points in the group receiving CSI alone and the group receiving combined RFA and CSI.

A linear mixed-effects model was constructed to evaluate the effects of group, time, and their interaction on VAS scores. There was no significant effect of age (β = −0.007, p = 0.311) or sex (β = −0.008, p = 0.966) on VAS scores. However, a significant main effect of group was observed (β = −0.948, 95% CI: −1.28 to −0.607, p < 0.001), indicating lower overall pain scores in the group receiving combined RFA and CSI compared to CSI alone. No significant between-group difference in VAS scores was observed at 1 week. A significant group × time interaction was observed at 3 months (β = 1.868, 95% CI: 1.492 to 2.244, p < 0.001) and 6 months (β = 0.965, 95% CI: 0.712 to 1.217, p < 0.001), indicating that VAS trajectories differed significantly between the groups at these follow-up points. The interaction at 1 week was not statistically significant (p = 0.529). The interaction plot demonstrated a similar trend between groups at the first week, followed by marked divergence at 3 and 6 months, with the group receiving combined RFA and CSI maintaining lower VAS scores over time compared to the group receiving CSI alone, which showed a decline in the initial treatment effect (Figure 4). Detailed results of the linear mixed-effects model are presented in Table 4.

## 4. Discussion

The main findings of this study demonstrate that integrating RFA with CSI yields significantly better outcomes, including sustained pain reduction and higher patient satisfaction, compared to CSI alone. While both interventions provided initial short-term improvement for patients with treatment-resistant PF, the combined protocol appears to be the key to bridging the gap between temporary relief and medium-term clinical stability.

Plantar fasciitis remains a persistent challenge in chronic pain management, and the lack of a definitive gold standard for refractory cases often leaves clinicians with limited options. Our data show that while CSI provides a robust antiinflammatory response, explaining the comparable VAS scores of the two groups during the first week, it lacks the durability to sustain that effect beyond the acute phase. At 3 and 6 months, the group receiving combined RFA and CSI maintained significantly lower VAS scores and higher GPE scores, whereas the CSI-alone group exhibited a clear “rebound” toward baseline pain levels. Linear mixed-effects model analysis revealed that, despite no significant difference being observed in the 1st week between the groups in the time-by-group interaction, the group receiving combined RFA and CSI maintained lower VAS scores at 3 and 6 months, whereas the CSI-alone group showed a reduction in the initial treatment effect over time.

Corticosteroid injection has been a standard treatment for PF since the 1950s [13]. This approach is favored for its cost-effectiveness, simplicity, and rapid pain relief, although potential complications may include hypersensitivity reactions, tendon rupture, and localized skin thinning [14]. Corticosteroid injection has shown a therapeutic success rate of about 90%, with its effects generally lasting up to 1 year [15]. However, these outcomes may vary depending on patient chronicity and are not always reproducible in treatment-resistant PF. Furthermore, a systematic review suggested that the benefits of CSI are often short-lived [16]. A recent metaanalysis indicated that while CSI effectively reduces heel pain in PF patients, its effects tend to be temporary, typically lasting 4–12 weeks [1]. This aligns with the results of previous studies, which showed that the pain scores of PF patients who received a steroid injection at the 6-month follow-up were nearly equivalent to their pretreatment levels [17,18]. Additionally, Rastegar et al. found that while steroid injections provide rapid relief of plantar heel pain, dry needling may offer more satisfactory long-term results [19]. Moreover, some studies have indicated that platelet-rich plasma injections may lead to greater improvements in pain relief and functional outcomes during the initial 3-month period compared to CSI [20,21]. In our study, the recurrence of pain observed in the CSI-only group reinforces the understanding of chronic PF as primarily a degenerative “fasciosis” rather than a simple inflammatory “itis.”

From an interventional pain management perspective, the superiority of the combined RFA and CSI approach is likely due to its dual-action mechanism. Since nerves mediate pain signals from any region of the body, RFA can be used to manage various etiologies of heel pain [22]. While corticosteroids address chemical inflammation, thermal RFA at 80 °C directly targets the sensory pathways of the medial and inferior calcaneal nerves. By achieving neurotomy of these nociceptive fibers, RFA effectively interrupts the chronic pain cycle. Furthermore, the localized thermal energy may induce a thermomodulatory effect that enhances microcirculation, potentially triggering a regenerative response in the degenerated fascia [9]. In addition to its neuromodulatory effects, RFA may also influence local tissue healing processes. Thermal energy generated during the procedure has been suggested to induce microvascular changes and the modulation of nociceptive signaling, potentially contributing to longer-lasting analgesia. Furthermore, targeting sensory branches such as the medial calcaneal or inferior calcaneal nerves may interrupt the chronic pain cycle associated with plantar fasciopathy. These mechanisms may partially explain the superior midterm outcomes observed in the group receiving combined RFA and CSI in our study.

Recent systematic reviews have also highlighted the effectiveness of radiofrequency-based techniques in the management of chronic plantar fasciopathy. Domingo-Marques et al. reported that radiofrequency microtenotomy using the Topaz technique significantly reduced pain and improved functional outcomes with low complication rates and high patient satisfaction in patients with refractory plantar fasciopathy [6]. Although the Topaz technique utilizes coblation-based radiofrequency and differs mechanistically from conventional thermal RFA, these findings support the broader role of radiofrequency-based minimally invasive interventions in the treatment of chronic plantar heel pain [6].

Our findings are supported by those of Yapici et al., who demonstrated the effectiveness of RFA in managing treatment-resistant cases, particularly in patients unresponsive to CSI or extracorporeal shockwave therapy [23]. In another study, RFA was found to be superior to CSI and the authors suggested that RFA is a safe and effective alternative when the long-term efficacy of steroids is limited [17]. Interestingly, while some authors reported no meaningful statistical differences in VAS scores at the 12th week [18], our study showed a distinct statistical advantage for the combined group at both 3 and 6 months (p < 0.001 for both). This divergence may be attributed to our specific combined technique, which simultaneously modulates both the inflammatory and neural components of the heel pain. In a recent study, Aktan and Aktan reported that combined ultrasound-guided CSI and RFA achieved superior long-term pain relief and functional improvement compared to either modality alone in recalcitrant PF [24]. These findings are consistent with our results and further support the benefit of targeting both inflammatory and neural components of PF.

Supporting this neural-targeted approach, Kırçiçek et al. demonstrated that ultrasound-guided pulsed radiofrequency (PRF) of the posterior tibial and sural nerves significantly reduced pain scores and improved functional capacity, as measured by American Orthopaedic Foot and Ankle Society scores, over a 6-month period [25]. The positive outcomes observed with both PRF of major nerve trunks and our specific targeting of distal sensory branches underscore the critical role of neuromodulation in the management of chronic heel pain.

The GPE scores in our RFA group remained consistently high, reflecting a meaningful improvement in functional capacity. This suggests that the clinical benefit of RFA is not merely a reduction in a score on a scale but a tangible improvement in daily mobility and quality of life that persists significantly longer than traditional injection therapies.

### 4.1. Limitations

The moderate sample size of this study may have limited the statistical power and generalizability. Additionally, the 6-month follow-up duration may not have adequately assessed the long-term efficacy of CSI alone or in combination with RFA, emphasizing the need for extended follow-up studies. Additionally, excluding patients with BMI of >30 kg/m^2^, despite obesity being a recognized risk factor for PF, may have limited the generalizability of our findings. The single-center design may have also limited the external validity of the findings. Another limitation of the present study is its retrospective design, which may have introduced potential selection bias.

While ultrasound-guided techniques are gaining popularity due to their portability and lack of ionizing radiation, our study reflects a period in which fluoroscopy was predominantly used. Fluoroscopy remains a valuable tool for ensuring precise needle positioning relative to skeletal structures, especially in retrospective studies evaluating the long-term outcomes of these established techniques.

## 5. Conclusion

Both CSI alone and combined RFA and CSI are effective minimally invasive options for achieving significant short-term pain relief in patients with chronic PF who have failed to respond to conservative management. However, our study highlights that the integration of thermal RFA into the injection protocol offers a clear clinical advantage in terms of durability and overall efficacy.

While the therapeutic effects of CSI tend to diminish by the 3rd and 6th months, the combined RFA and CSI approach provides superior medium-term pain control and greater patient-reported global improvement. Based on these findings, the addition of RFA to CSI may provide improved medium-term pain relief and greater patient-reported clinical improvement in patients with refractory PF. Further prospective studies with longer follow-up are warranted to confirm these findings.

## Figures and Tables

**Table 1:** Demographic characteristics of the treatment groups.

	Steroid injection group (n=78)	Combined RFA + CSI group (n=100)	p-value	Cohen’s d	95% CI for Cohen’s d	
					Lower	Upper
**Age (years) mean ± SD**	52.5 ± 10.7	53.6 ± 10.7	0.878*	0.037	−0.260	0.333
**Sex (n/%)**			0.990†			
**Female**	60 (76.9)	77 (77.0)				
**Male**	18 (23.1)	23 (23.0)				

* Independent-samples t-test;

† Pearson chi-square test;

CI: confidence interval; RFA: radiofrequency ablation, CSI: corticosteroid injection.

**Table 2:** Visual analog scale and global perceived effect scores of the groups during follow-up.

	Steroid injection group (n=78)		Combined RFA + CSI group (n=100)		p-value*	95% CI	
	Median (IQR)	Median (min–max)	Median (IQR)	Median (min–max)		Lower	Upper
VAS-baseline	7.0 (1.3)	7.0 (5.0–9.0)	7.0 (1.0)	7.0 (5.0–9.0)	0.057	0.020	0.067
VAS-1st week	3.0 (3.0)	3.0 (0.0–8.0)	3.0 (2.0)	3.0 (1.0–9.0)	0.447	0.367	0.526
P†	<0.001		<0.001				
VAS-3rd month	4.0 (3.0)	4.0 (1.0–8.0)	2.0 (1.8)	2.0 (1.0–9.0)	<0.001	0.000	0.020
P†	<0.001		<0.001				
VAS-6th month	5.0 (2.0)	5.0 (2.0–8.0)	2.0 (1.0)	2.0 (1.0–9.0)	<0.001	0.000	0.020
P†	<0.001		<0.001				
GPE-1st week	6.0 (2.0)	6.0 (4.0–7.0)	6.0 (1.0)	6.0 (4.0–7.0)	0.100	0.052	0.148
GPE-3rd month	5.0 (2.0)	5.0 (3.0–6.0)	6.0 (1.8)	6.0 (3.0–7.0)	<0.001	0.000	0.020
GPE-6th month	5.0 (2.0)	5.0 (3.0–6.0)	6.0 (3.0)	6.0 (4.0–7.0)	<0.001	0.000	0.020

* Mann–Whitney U test and false discovery rate correction;

† p-values indicate difference from baseline for VAS scores according to the Wilcoxon signed-rank test;

CI: confidence interval; VAS: visual analog scale, GPE: global perceived effect. Confidence intervals represent the estimated ranges of the between-group differences.

**Table 3:** Meaningful pain relief in treatment groups during follow-up.

	Steroid injection group (n=78)	Combined RFA + CSI group (n=100)	p-value*
**Meaningful pain relief ratio**			
1st week	54 (69.2)	71 (71.0)	0.798
3rd month	23 (29.5)	83 (83.0)	<0.001
6th month	12 (15.4)	87 (87.0)	<0.001

* Pearson chi-square test;

RFA: radiofrequency ablation, CSI: corticosteroid injection.

**Table 4:** Linear mixed-effects model adjusted for age and sex.

Parameter	Beta	SE	95% CI		p-value
			Lower	Upper	
Intercept	4.947	0.129	4.69	5.201	<0.001
Age	−0.007	0.007	−0.02	0.007	0.311
Sex	−0.008	0.198	−0.399	0.382	0.966
Group (CSI alone vs combined RFA and CSI)	−0.948	0.172	−1.28	−0.607	<0.001
Time (1 week)	3.974	0.196	3.58	4.361	<0.001
Time (3 months)	−1.448	0.143	−1.73	−1.166	<0.001
Time (6 months)	−0.705	0.096	−0.89	−0.515	<0.001
Group × time (1 week)	0.165	0.262	−0.350	0.681	0.529
Group × time (3 months)	1.868	0.191	1.492	2.244	<0.001
Group × time (6 months)	0.965	0.128	0.712	1.217	<0.001

SE: Standard error; CI: confidence interval; CSI: corticosteroid injection; RFA: radiofrequency ablation.
